# Biopsy for Suspicious Oral Lesions: A Review From the American Head and Neck Society‐Cancer Prevention Service

**DOI:** 10.1002/hed.70148

**Published:** 2025-12-30

**Authors:** James Christopher Gates, Heather Edwards, Alessandro Villa, Nick Purdy, Michael Troka, Peter Varela, Quinn Self, Yingci Liu, Yusuf Dundar, Patricia Joyce Brooks, Dauren Adilbay, Andrew Birkeland, John Cramer

**Affiliations:** ^1^ Department of Oral and Maxillofacial Surgery Hospital of the University of Pennsylvania Philadelphia Pennsylvania USA; ^2^ Boston University Chobanian and Avedisian School of Medicine Boston Massachusetts USA; ^3^ Department of Surgery Director of Oral Oncology, Baptist Health Miami Florida USA; ^4^ Department of Otolaryngology Geisinger Health System Danville Pennsylvania USA; ^5^ University of Pennsylvania Philadelphia Pennsylvania USA; ^6^ Department of Otolaryngology University of Vermont Burlington Vermont USA; ^7^ Department of Diagnostic Sciences Rutgers School of Dental Medicine Newark New Jersey USA; ^8^ Department of Otolaryngology Texas Tech University Lubbock Texas USA; ^9^ Princess Margaret Cancer Centre Toronto Ontario Canada; ^10^ Department of Otolaryngology Medical University of South Carolina Charleston South Carolina USA; ^11^ Department of Otolaryngology UC Davis Sacramento California USA; ^12^ Department of Otolaryngology Wayne State University Detroit Michigan USA

## Abstract

**Background:**

Oral cancer is often preceded by a precursor lesion. This presents an opportunity for early diagnosis and intervention. Method of biopsy and interpretation are not well standardized and novel methods of analysis are now being investigated.

**Methods:**

We conducted a *narrative review* of PubMed/MEDLINE (last search August 31, 2025), focusing on adult *oral precancerous lesions* evaluated in outpatient settings.

**Results:**

Incisional punch biopsy is reproducible and often provides the diagnostic information needed. However, scalpel biopsy should be considered when initial biopsy is equivocal, depth of invasion is desired, or to minimize sampling bias. Limited studies show improved sensitivity of combining saliva and plasma sampling. Targeted fluorescent imaging may aid in future biopsy site selection. AI has shown encouraging results in both automated detection of dysplasia and prediction of malignant progression, achieving performance comparable to clinically validated grading systems.

**Conclusion:**

This update serves to further inform biopsy of oral suspicious lesions and provide a framework for future investigation.

## 1 Background/Introduction

Head and neck cancer is a global concern. In 2022, GLOBOCAN estimated that there were over 700 000 new head and neck cancer diagnoses (including cancers of the lip, oral cavity, nasopharynx, oropharynx, and hypopharynx), and over 300 000 deaths worldwide [1]. Early detection has the potential to improve outcomes. Notably, malignant oral lesions often have clinically identifiable precursor lesions such as leukoplakia or erythroplakia. This provides a potential diagnostic window of opportunity. However, obtaining a timely biopsy and, in turn, diagnosis and treatment remains a challenge for some patients.

Malignancy can advance during lag time from recognition of the lesion to diagnosis and treatment. Longer wait times, or delay in diagnosis, have been shown to worsen the prognosis of patients with oral squamous cell carcinoma (OSCC) [2]. Improving techniques and access to biopsy could help to reduce time to treatment.

Comparative data on the diagnostic performance of *specimen‐collection techniques* (scalpel, punch, brush) for oral premalignant (also referred to as oral potentially malignant disorders or precancerous lesions) and malignant lesions are limited. Since the era when brush biopsy was primarily cytology‐based, *adjunct molecular methods* and *AI‐assisted analytics* have emerged and may enhance triage accuracy; however, they *do not replace* histopathology and remain adjuncts in most settings. *Liquid biopsy* (saliva/plasma) is under active investigation for diagnosis and disease monitoring but is not yet standard for oral lesions. These technologies *could broaden access to initial evaluation* in general practice. For these reasons, the American Head and Neck Society (AHNS) Cancer Prevention Service undertook a *narrative review* to summarize current evidence and practical considerations for biopsy of oral cavity precancerous and cancerous lesions.

## 2 Methods

We conducted a *narrative review* of PubMed/MEDLINE (last search August 31, 2025), focusing on adult *oral precancerous lesions* evaluated in outpatient settings. Search terms included combinations of *oral leukoplakia/erythroplakia*, *oral squamous cell carcinoma*, *biopsy* (scalpel/punch/brush), *cytology*, *liquid biopsy*, *fluorescence imaging*, *molecular testing*, and *artificial intelligence*. We prioritized *systematic reviews, randomized or prospective studies*, and *large observational cohorts*; smaller series were included where higher‐level evidence was lacking. This is *not* a systematic review; study selection and data abstraction were performed by domain experts, and conclusions emphasize the *consistency of findings* across sources rather than pooled estimates.

## 3 Results

### 3.1 Specimen Collection Techniques

#### 3.1.1 Incisional Biopsy

There are several ways to perform an incisional type of biopsy, but most common among those treating oral premalignant lesions are: punch, scalpel, and brush biopsy. There are advantages and disadvantages to each (Table 1). From a technical perspective, all biopsies are performed after injection of local anesthesia typically with a vasoconstrictor. For punch biopsy, a 3‐, 4‐, or 5‐mm punch is used to outline an incision through epithelium, sub‐epithelial layer, to muscle. Next, a tooth pickup and scissors are used to elevate and excise the index tissue at the base of the lesion, incorporating sub‐epithelial tissue. The base is cauterized and typically left to heal secondarily. For brush or swab biopsy, the brush is lightly applied directly to the oral lesion with just enough pressure so that the handle of the brush bows slightly. Next, the brush is rotated 360° on the surface of the lesion to collect cellular material. If the tissue is to be fixed immediately, it is placed at one edge of a new glass slide and again rolled 360° over its surface to deposit cellular contents on the slide. For scalpel biopsy, a blade, typically #15, is held like a pen, with the blade perpendicular to the tissue plane and the belly of the blade used to incise either an elliptical or box‐shaped incision in the index lesion. This, like punch biopsy, is carried through epithelium to the subepithelial or muscular plane, and then the lesion is excised in this plane with scissors or a blade at an angle staying above muscle and closed with sutures. Thus, it is evident that punch and scalpel biopsy can obtain a depth past the epithelium, to detect invasion, whereas brush biopsy only collects superficial epithelial cells (Table 1).

**TABLE 1:** Comparative characteristics of oral lesion biopsy modalities.

Characteristic	Scalpel incisional biopsy	Punch biopsy	Brush cytology (oral brush)
Sampling depth and architecture	Full‐thickness mucosa; best preservation of tissue architecture; facilitates depth of invasion (DOI) assessment	Full‐thickness mucosa for small lesions; good architecture in most cases	Superficial epithelial cells; no architecture; cannot assess invasion
Typical role	Reference diagnostic standard; used when invasion/DOI assessment is needed or initial biopsy is equivocal	Common first‐line for straightforward lesions; reproducible; small defects	Adjunctive/triage test; abnormal results should trigger tissue biopsy
Anesthesia and setting	Local ± OR for larger lesions	Local, office‐based	Often no local; office‐based
Complications (relative)	Pain, bleeding, infection, delayed healing (higher than punch for many sites)	Generally minor pain/bleeding; small wounds	Minimal procedure‐related risk; risk of false positives/negatives leading to additional procedures
Advantages	Gold‐standard histology; margins/architecture; DOI	Fast; inexpensive; reproducible	Minimally invasive; easy sampling; potential cost savings as a triage step
Key limitations	Larger wound; suturing; operator variation	Limited specimen size in thick/keratinized sites; may miss invasion in select cases	Lacks architecture; performance varies by technique/lab; payer coverage variable; not a replacement for tissue histology
Evidence anchors	ADA guideline treats scalpel histopathology as reference standard	Meta‐analysis suggests fewer artifacts than scalpel in some series; widely used	2021 Cochrane: sensitivity ~0.90, specificity ~0.94 as adjunct—moderate certainty

Recently, the AHNS cancer prevention service performed a survey of otolaryngologists and oral surgeons, published in 2023. Results from this survey demonstrated a strong preference in all respondents for incisional punch biopsy as the initial tissue‐sampling technique for oral lesions (85.1% of respondents across all demographics). Scraping/scalpel biopsy was only used in 1.5% of respondents, and brush cytology in 3.0% [3].

Survey data suggest a *preference for punch biopsy* as an initial technique in many practices [3]. A meta‐analysis comparing *tissue artifacts* reported higher rates of crush, split, and fragmentation artifacts with *scalpel* versus *punch* biopsies, although *diagnostic sensitivity/specificity and cost* were not directly compared [4]. In routine settings, punch biopsy is favored for *ease of use*, *anatomic preservation*, and *reproducibility*.

*Scalpel incisional biopsy* remains fundamental when *depth of invasion (DOI)* or broader *architectural assessment* is needed and is treated as the *reference diagnostic standard* in the American Dental Association (ADA) guideline [5]. ADA guidelines make this recommendation to ensure that suspicious lesions identified on screening via exam and brush biopsy are subsequently sampled with greater depth than brush biopsy. For this reason, they recommend scalpel biopsy as the gold standard. Therefore, inherent in their recommendation is the importance of a more definitive, deeper biopsy, and less a comparison of scalpel to punch biopsy.

In regard to complications and side effects, scalpel biopsy may entail *greater local morbidity* (pain, bleeding, delayed healing) in some sites [6, 7, 8, 9, 10]. *Technique selection* should be driven by anticipated diagnostic needs (e.g., DOI), lesion location, and patient factors.

Reliable correlation has been demonstrated between incisional biopsy and final diagnosis. A study by Chen et al. that included 272 patients reported a concordance rate of 88.9% between incisional biopsy and final excisional pathology. Among discordant cases, the most common cause was sampling error (60%), followed by interpretive discrepancies (23.3%) and inadequate or inflammatory artifacts.

Most methods of biopsy can be used for either incisional or excisional biopsy, depending on the size of the lesion. The methods most commonly used for incisional biopsy are punch, scalpel, or brush. However, if the lesion is small and a punch biopsy is 5 mm, exceeding the diameter of the lesion (3 or 4 mm) then the punch would be considered excisional. The same is true for a scalpel, which could be used to incise or excise the lesion. Therefore, the technique doesn't determine the nature of the biopsy, but rather the amount of lesion excised does. Finally, more important than the technique chosen is the decision whether to take a small piece of the lesion, which preserves the architecture of the lesion as well as the margins, or to excise the entire lesion, which leaves only scar behind.

While incisional biopsy is highly effective, its accuracy hinges on thoughtful site selection to minimize sampling bias, adequate specimen size, and clear clinical‐pathologic communication. Punch biopsy is reproducible and often provides diagnostic information needed. However, scalpel biopsy should be considered when initial biopsy is equivocal or DOI is desired. In addition, when multiple incisional biopsies do not yield a definitive diagnosis or are discordant with the clinical diagnosis or gestalt of the provider, consideration should be given for excisional biopsy (Table 1).

#### 3.1.2 Excisional Biopsy

Excisional biopsy offers certain advantages over incisional biopsy. Errors may occur if incisional biopsies are taken from necrotic or unrepresentative areas or contain insufficient tissue. In excisional biopsy, larger biopsy volume was significantly associated with higher diagnostic accuracy (mean 1.53 vs. 0.42 cm^3^, *p* < 0.01), highlighting the importance of adequate tissue sampling [8].

However, excisional biopsy also carries disadvantages. Compared with excisional biopsy, incisional biopsy has been found to preserve margins of the lesion for subsequent oncologic treatment planning and re‐excision. For example, if incisional biopsy yields a high grade or invasive lesion, measurements can be taken from the intact lesion margins to re‐excise it with an appropriate wide oncologic margin. When lesions are excised at initial biopsy, the architecture is disturbed and visual margins of the index lesion no longer exist. In addition, there is scarring and erythema in early healing that can mimic a high grade lesion. The loss of margins, disturbed architecture, and scarring can confound subsequent treatment planning and result in under or overtreatment. For this reason, excisional biopsy can counterintuitively lead to less oncologic predictability in subsequent re‐excision if high grade/invasive and if not originally done with oncologic intent. Retrospective analysis of excisional biopsy for early‐stage oral cavity cancer, as compared to oncologic excision, was found to present less valuable histologic data. In their study, Schemel et al. found that equivocal data from such excisional biopsy led to lower rates of neck dissection, adjuvant radiation therapy, and overall worse local control as compared to incisional biopsy and subsequent oncologic excision [11].

For these reasons, incisional punch biopsy is the overwhelming favorite initial method chosen by head and neck surgeons [3]. In most instances, one or a series of punch biopsies yields the information needed and only rarely should a lesion need to be excised if initial incisional biopsies do not yield a diagnosis or are discordant with the incisional diagnosis.

While excisional biopsy provides optimal tissue volume for diagnosis to minimize sampling bias and provides DOI, it may also be associated with decreased oncologic data as compared to excision with oncologic margin and greater patient discomfort than incisional techniques.

In cases where initial histopathology is non‐diagnostic, discordant, or equivocal, but clinical suspicion remains high, re‐biopsy or complete excision should be considered.

#### 3.1.3 Brush Biopsy

Brush biopsies offer many advantages for both the patient and provider. For the provider, the technique is minimally invasive, enabling samples to be easily collected in‐office without the need for local anesthesia, suturing, or additional training. Patients, particularly those with dental or needle phobia, may experience anxiety reduction during the procedure.

Across heterogeneous techniques and reference standards, reported *accuracy of brush cytology* is variable. The highest level of evidence, a Cochrane review, shows correlation (~0.90 and specificity ~0.94) between brush biopsy and clinical diagnosis with moderate certainty (Table 1). However, the recommendation is for use as an adjunct, not comparable or to replace diagnostic accuracy of histology [12]. Concordance with tissue diagnosis can be high in selected settings, and *cost modeling* suggests potential savings when used to triage lesions prior to tissue biopsy [13].

Additional studies of lower‐level evidence show high sensitivity and specificity exist when comparing brush biopsy to other methods from a cytologic perspective (Table 1) [14, 15]. Cytological analysis is heavily reliant on the pathologist's experience and skill in analyzing the biopsy, which can cause variability in interpretation. Moreover, the diagnosis of dysplasia and invasive carcinoma is made not just from cytology but from its relationship to the epithelial and stromal architecture, respectively. Therefore, the most important shortcoming of brush biopsy is a lack of diagnostic information related to architecture, which is necessary to grade dysplasia and diagnose invasive carcinoma. This has limited its utility in clinical practice. Commercial laboratory services (e.g., OralCDx‐type platforms) are available; *coverage and performance* may vary by technique and laboratory. Current evidence supports its use primarily for *triage*, with *abnormal results prompting tissue biopsy* for definitive diagnosis [12, 16].

#### 3.1.4 Liquid Biopsy

Liquid biopsies are biofluid‐based tools that enable non‐invasive detection of disease for purposes such as diagnosis, surveillance, and monitoring treatment response. Tumors shed circulating tumor cells, extracellular vesicles (EV), and cell‐free DNA (cfDNA), termed circulating tumor DNA (ctDNA) into nearby biofluids, which can be analyzed for clinically relevant biomarkers [17, 18, 19, 20]. Fluid‐based sampling, such as from plasma or saliva, can overcome limitations of tissue biopsies, including geographic sampling errors and issues with tumor accessibility.

In contrast to the viral‐driven diseases, the molecular heterogeneity of oral cancer presents challenges for implementing tumor‐naïve detection strategies [21]. Additionally, the proximity of lesions to the fluid source may present a limitation for early‐stage disease. To overcome this challenge, multiple fluid sources can be obtained. For example, Wang et al. assessed multi‐source liquid biopsies in the detection of mutations in HNSCC patients for head and neck squamous cell carcinoma (SCC), including OSCC. In this observational study, cfDNA from plasma and total salivary DNA was used for the detection of HPV DNA along with somatic mutations using multiplex PCR to target potential oncogenes. Plasma alone had a sensitivity of 87%, while saliva alone had a sensitivity of 76%; when combined, the sensitivity improved to 96%. Most interestingly, salivary DNA gave 100% detection sensitivity in OSCC patients specifically, highlighting the value of liquid biopsy source proximity and especially those studies in saliva [22].

Tumor‐informed detection strategies have also been employed as a means for designing personalized bespoke ctDNA assays [23]. HPV‐negative HNSCC patient plasma was interrogated following tumor whole‐exome sequencing for personalized variant selection, achieving 100% sensitivity. In all patients that recurred, ctDNA was detected prior to clinically observed progression, with lead times greater than 3 months [23]. Similarly, Sans Garcia et al. obtained higher sensitivity with a tumor‐informed bespoke ctDNA plasma assay when compared to tumor‐naïve panels such as CAPP‐seq, [24] indicating that knowledge of mutational targets understandably increases the detection rate. Honore et al. similarly observed lower sensitivity using a tumor‐agnostic 26‐gene NGS panel looking at HNSCC plasma [25]. Tumor‐informed mutational profiles were also utilized to assay the ability to detect disease in salivary rinses of patients with HNSCC with NGS and found to have sensitivity of 95.9% [26]. These works show that tumor‐informed mutation‐based ctDNA assays more accurately detect disease in non‐viral HNSCC; however, they are more complex and costly and obviously are not useful for tumor‐naïve cancer screening approaches.

Many cancers, including OSCC, show more instances of epigenetic changes such as hypermethylation than genetically mutated or altered DNA abnormalities, providing an avenue for greater sensitivity for ctDNA detection [27]. Burgener et al. examined cell‐free Methylated DNA ImmunoPrecipitation and high throughput sequencing (cfMeDIP‐seq) together with mutation‐based NGS from plasma cfDNA HPV‐negative HNSCC patient plasma [28]. Pre‐treatment ctDNA levels were highly correlated with disease, and persistent post‐treatment levels of methylated ctDNA were strongly associated with recurrence. These results suggest that the value of epigenetic profiles of cfDNA can support the detection of disease along with mutational profiling. A larger cohort of patients consisting of both viral and non‐viral‐associated HNSCCs showed similar findings, indicating the translational opportunities this strategy holds [29].

Overall, in the absence of tissue, analysis of multiple biofluid sources could provide a means for driving greater sensitivity of disease in oral cancer detection. Recent advancements in sequencing technologies have enabled the identification of biomarkers in various fluid types using a range of targeted methods. These techniques hold *investigational potential* for earlier diagnosis, minimal residual disease (MRD) detection, and response monitoring in oral cancer; *validation in prospective cohorts* with standardized endpoints is needed. However, no FDA‐cleared/approved liquid biopsy is indicated for OSCC diagnosis or MRD; platforms used in other cancers do not have HNSCC indications (Table 2).

**TABLE 2:** Technology and regulatory status of selected adjuncts (United States).

Technology/agent	Typical setting	Intended use (current practice)	US FDA status (as of September 16, 2025)	Notes/examples
Adjunctive tissue autofluorescence/chemiluminescence devices (e.g., VELscope, Identafi/ViziLite families)	Dental/ENT clinic	Adjunctive visualization to highlight mucosal change; not diagnostic alone	510 (k)‐cleared as adjunctive oral mucosal examination aids (e.g., VELscope K060920; subsequent modifications K070523)	Labeling emphasizes adjunct use and need for clinical judgment/biopsy when indicated.
Targeted fluorescent imaging (TFI) (e.g., panitumumab‐IRDye800CW, PARPi‐FL)	OR/clinic under protocols	Image contrast for lesion/margin visualization; biopsy guidance	Investigational; no FDA marketing authorization for oral cancer detection/biopsy guidance	Panitumumab‐IRDye800CW and PARPi‐FL reported in early‐phase trials; conducted under IND/clinical trials.
Brush cytology (oral brush) with lab analysis (e.g., OralCDx‐type services)	Clinic → CLIA lab	Adjunct/triage cytology; abnormal results prompt tissue biopsy	Commercially available lab services; no publicly listed FDA IVD clearance that replaces tissue histology	Performance best viewed as adjunct; payer policies vary; tissue biopsy remains definitive.
Liquid biopsy for OSCC/HNSCC (saliva/plasma ctDNA, methylation, EVs)	Clinic → specialized labs	Detection/monitoring/MRD (research and limited clinical use)	No FDA‐cleared/approved assay specifically for OSCC/HNSCC diagnosis or MRD	FDA‐approved liquid CDx exist for other cancers (e.g., Guardant360 CDx, FoundationOne Liquid CDx), not for HNSCC indications.
HPV tests for mouth/throat	—	Screening/diagnosis of oral/oropharyngeal HPV	No FDA‐approved test for detecting HPV in the mouth or throat	Cervical HPV tests are FDA‐approved (including self‐collection pathways), but not for oral sites.
AI/ML for oral lesion diagnosis (photos/brush/histology)	Clinic/lab	Automated detection/triage	No FDA‐cleared AI for diagnosing oral epithelial dysplasia/cancer	FDA‐cleared dental AI exists for radiograph analysis (2D/3D) but not for oral mucosal cancer diagnosis.

### 3.2 Specimen Site Selection/Targeting Methods

#### 3.2.1 Targeted Fluorescent Imaging

Selecting the optimal biopsy site in multifocal, extensive, or clinically ambiguous lesions remains a significant challenge [30]. Conventional guidance tools such as visual inspection, toluidine blue, and autofluorescence imaging lack molecular specificity and often fail to identify the most biologically aggressive regions [30, 31, 32].

Targeted fluorescent imaging is emerging as a transformative adjunct for both diagnostic risk stratification and biopsy guidance [33]. This approach employs systemically or topically administered imaging agents—such as fluorescently labeled antibodies, small‐molecule ligands, or proteins—that selectively bind to cell surface or nuclear biomarkers upregulated during dysplastic transformation. In the context of OPLs, targets such as Programmed Death‐Ligand 1 (PD‐L1), Mesenchymal–Epithelial Transition factor (MET), NOTCH1, and podoplanin have shown differential expression in high‐risk lesions and are under active investigation for multiplexed, multispectral imaging strategies [34, 35, 36, 37]. These markers not only reflect tumor biology but also offer the potential to guide biologically informed, site‐specific biopsies, reducing sampling errors and increasing diagnostic yield.

Clinical progress in targeted imaging for oral cancer surgery underscores the potential of this approach. EGFR‐targeted agents, such as panitumumab‐IRDye800CW, have demonstrated safety and efficacy in Phase I and II trials for head and neck SCC [38, 39]. In these studies, intraoperative fluorescent imaging improved the detection of residual tumor at deep and peripheral margins with 100% sensitivity for identifying positive margins and 70.3% for close margins. Similarly, PARP1‐targeted imaging using PARPi‐FL (a fluorescent analog of olaparib) has been explored for topical application in early oral cancer, with high tumor‐to‐background contrast and specificity for dysplastic epithelium [33]. In a recent clinical feasibility study, topical PARPi‐FL accurately delineated OSCC lesions within minutes, supporting its potential utility in point‐of‐care visualization without the need for intravenous injection [33].

Early clinical studies demonstrate *feasibility and safety in oral cancer*, including improved intraoperative visualization in selected cohorts [33, 38, 39]. *EGFR‐ and PARP‐targeted agents remain investigational* and currently *lack FDA marketing authorization* for oral lesion detection or margin assessment (Table 2).

### 3.3 Specimen Interpretation

#### 3.3.1 Molecular Testing

Tissue biopsy with histological analysis remains the gold standard for diagnosing malignant and precancerous lesions in the head and neck. Given the invasive, sometimes subjective and laborious nature of traditional tissue diagnosis, alternative techniques are desirable. Advancements in molecular testing may *augment* minimally invasive techniques (brush, liquid biopsy) as *adjuncts* to histology in research and select clinical contexts. These platforms carry the potential for low‐risk screening, diagnosis, and treatment response tests in head and neck malignancies (Table 2) [40].

Advancing these technologies first requires identification of reliable molecular signatures that can differentiate between normal, premalignant, and malignant lesions. HNSCC makes up the lion's share of head and neck malignancies. As such, numerous molecular signatures of HNSCC are being studied for clinical application.

In non‐HPV mediated HNSCC, there is mutational heterogeneity; therefore, a wide array of somatic mutations must be analyzed. In non‐HPV HNSCC, *mutational heterogeneity* necessitates broad panels (e.g., *TP53, PIK3CA, CDKN2A, FBXW7, HRAS/NRAS*). *Tumor‐informed* assays can achieve high detection rates in OSCC tissue and *saliva/plasma* [24], whereas *tumor‐naïve* approaches show *lower sensitivity* across head and neck subsites. Routine *diagnostic* use for oral lesions has *not* been established. Despite significant promise, routine application in non‐HPV mediated HNSCC has not been established.

Testing epigenetic alterations to DNA is also an area of focus. Methylation signatures influence gene expression such as that of TP53 and can be an early marker of tumorigenesis, making this a particularly attractive target in pre‐malignant lesions. Methylation can be tested on traditional specimens as well as in plasma and saliva; however, there is significant heterogeneity in methylation signatures, and a broad sequence of the genome needs to be analyzed to increase test accuracy. Although promising, clinically applicable testing remains in development [29, 41].

Other molecular tests are in development to detect changes within malignant cells resulting from somatic changes. Although specificity may be lower, altered gene expression should precede phenotypic changes, making this useful in pre‐malignant identification. Messenger RNA is one such target for evaluation. To date, mRNA changes have also been clinically correlated with disease response to treatment; however, broad application has not been widely employed [41].

EV also offer a promising target for testing. They contain a complex milieu of proteins, DNA, messenger‐, and micro‐RNA as well as metabolites and are critical for intercellular messaging. Their surface protein signatures and the micro‐RNA within them have been correlated to disease burden and response to treatment. Although an active field of study, these are particularly challenging to study given the inherent complexity of isolating and testing vesicles [42].

Finally, metabolomic, lipidomic, and proteomic signatures of patients with HNSCC are being evaluated. While changes in these profiles correlate with disease status, there is significant limitation, particularly in the specificity of these tests given the extensive heterogeneity and multifactorial influence of upstream signals [42].

Taken as a whole, there has been significant recent progress in the identification and validation of molecular markers for head and neck cancers. Viral DNA testing has already taken a significant foothold in the treatment of virally mediated tumors. Other molecular markers offer the promise of developing screening, diagnostic, and surveillance testing for managing non‐viral head and neck malignancies.

These advancements demonstrate the need to move beyond static biomarkers to better identify high‐risk oral lesions. Advanced profiling of oral premalignant lesions through PCR analysis, whole exome, and RNA sequencing has been done in small trials. Additional multi‐site trials should be instituted to better identify progressing versus non‐progressing patterns in these lesions and identify potential targets for therapy. *At present*, molecular testing for oral lesions (mutations, methylation, EVs, mRNA) should be regarded as *adjunctive or research‐stage*; *histology remains the diagnostic standard*.

#### 3.3.2 Artificial Intelligence and Machine Learning

Artificial intelligence (AI) has emerged as a powerful tool in the early detection and histopathologic evaluation of precancerous oral cavity lesions. The integration of AI into medical imaging typically involves three primary stages: preprocessing, segmentation, and postprocessing [43]. Preprocessing enhances image quality by eliminating unwanted data and minimizing noise, while segmentation focuses on accurately delineating regions of interest, such as differentiating pathological areas from healthy tissue. Postprocessing techniques, including the use of convolutional neural networks (CNNs), enable the extraction of critical features such as lesion borders and architectural patterns [43]. AI‐assisted approaches have shown *promising discrimination* between abnormal and normal mucosa, with *higher performance* reported when paired with OCT in small studies and meta‐analyses. A recent systematic review and meta‐analysis of 12 studies reported a diagnostic odds ratio (DOR) of 121.66 (95% confidence interval [CI]: 29.60–500.05) for AI‐supported screening, with subgroup analysis indicating superior diagnostic performance for OCT (324.33 vs. 66.81 and 27.63) and negative predictive value (0.94 vs. 0.93 and 0.84), compared to photographic imaging and autofluorescence [44]. *Access to OCT* remains limited outside major centers; *generalizability* across devices and care settings requires prospective validation.

Despite the promise of emerging technologies, histopathologic evaluation remains the current gold standard for assessing the risk of malignant transformation in oral potentially malignant disorders. Microscopically, lesions are graded for the presence and severity of epithelial dysplasia (mild, moderate, severe) in accordance with WHO guidelines [45], with a higher grade strongly correlated with increased risk of developing oral cancer [46, 47]. While universally applied in biopsy assessment, dysplasia grading relies on a spectrum of cytologic and architectural features that are inherently subjective, leading to high interobserver variability, even among experienced pathologists [48, 49]. This variability underscores the appeal of AI models in digital pathology, where computational tools can enhance accuracy and reproducibility.

To this end, AI has shown encouraging results in both automated detection of dysplasia and prediction of malignant progression, achieving performance comparable to clinically validated grading systems. For detection of dysplastic lesions on histopathology, reported accuracies/F1‐scores range from 90.6%–96%, sensitivities of 93.0%–96.5%, and precisions of 74.0%–97.0% [50, 51, 52, 53]. For prediction of malignant transformation, accuracies/AUROCs of 0.71–0.77 [52, 53, 54] have been described. Nonetheless, further progress is needed, as distinguishing between progressive and non‐progressive dysplasia is a subtle and difficult visual task, compounded by the scarcity of publicly available, well‐annotated datasets for model training. Recent advances in computer vision provide opportunities for more powerful AI models beyond traditional CNNs. Vision Transformers (ViTs) and Multiple Instance Learning (MIL) frameworks can capture complex, correlated image features with greater fidelity, making them particularly relevant for pathology tasks [50, 55, 56, 57].

Finally, image‐based models alone cannot capture the full complexity of an individual patient's risk. Multimodal AI approaches that integrate histopathology with clinical, demographic, laboratory, and imaging data to achieve more comprehensive prediction are increasingly recognized as essential in medicine. For oral potentially malignant disorders, this means combining histologic features with patient‐specific risk factors such as age, immune status, tobacco and alcohol history, and lesion site, as these clinical factors are known to influence malignant transformation risk. However, only a limited number of studies have explored this multimodal approach to date [58], making it a ripe area for future research.

Beyond imaging, AI models applied to *histopathology* report high accuracy in retrospective series [45]. *FDA‐cleared AI* exists for *dental radiographs* and *cervical cytology*, but *no FDA‐cleared AI* currently diagnoses *oral epithelial dysplasia/cancer* from oral images or brush cytology (Table 2). Early results are encouraging, yet *clinical utility* depends on *prospective, multi‐site validation* with patient‐important endpoints.

The continued advancement of AI applications offers new opportunities to enhance screening and diagnostic strategies for oral lesions. The development and widespread incorporation of AI into histopathology, brush biopsy/cytopathology may further improve access to early detection, reduce delays in care, and support timely triage and treatment, particularly for high‐risk and underserved populations in rural or remote areas.

## 4 Conclusion

Incisional punch biopsy is reproducible and often provides the diagnostic information needed. However, scalpel biopsy should be considered when initial biopsy is equivocal, or DOI is desired. Excisional biopsy provides optimal tissue volume for diagnosis to minimize sampling bias; however, it may also be associated with decreased oncologic data as compared to excision with oncologic margin and greater patient discomfort than incisional techniques. In the United States, brush cytology functions as an *adjunctive* tool; *abnormal* or *indeterminate* findings should be followed by *scalpel or punch* biopsy. Utility of liquid biopsy for OSCC diagnosis is of great interest. In limited cohorts, combining saliva and plasma improved sensitivity of cancer detection. Currently, no FDA‐cleared/approved liquid biopsy modality is indicated for OSCC diagnosis; however, justification exists to pursue such an end.

Targeted fluorescent shows promise for biopsy site selection. However, these agents remain *investigational* and currently *lack FDA marketing authorization* for oral lesion detection or margin assessment. Molecular advancements demonstrate the need to move beyond static biomarkers to better identify high‐risk oral lesions. Multi‐site trials should be instituted to better identify progressing vs. non‐progressing molecular patterns in these lesions, investigating molecular tools.

AI has shown encouraging results in both automated detection of dysplasia and prediction of malignant progression, achieving performance comparable to clinically validated grading systems. Nonetheless, further progress is needed, as distinguishing between progressive and non‐progressive dysplasia is a subtle and difficult visual task. This is compounded by the scarcity of publicly available, well‐annotated datasets for model training. The development and widespread incorporation of AI into histopathology and brush biopsy/cytopathology may further improve access to early detection, reduce delays in care, and support timely triage and treatment, particularly for high‐risk and underserved populations.

## Funding

The authors have nothing to report.

## Data Availability

The authors have nothing to report.
